# Pfs47 as a Malaria Transmission-Blocking Vaccine Target

**DOI:** 10.4269/ajtmh.21-1325

**Published:** 2022-07-11

**Authors:** Alvaro Molina-Cruz, Carolina Barillas-Mury

**Affiliations:** Laboratory of Malaria and Vector Research, National Institute of Allergy and Infectious Diseases, National Institutes of Health, Rockville, Maryland

## Abstract

Transmission-blocking vaccines (TBVs), pioneered by Richard Carter and others, aim to prevent parasite development in the mosquito vector and are a promising new tool for malaria elimination. Pfs47, recently identified as a TBV target, is a three-domain 6-cysteine protein on the surface of *Plasmodium falciparum* sexual stages. Pfs47 allows the parasite to evade mosquito immunity and is key for *P. falciparum* infection of the dominant malaria vectors *Anopheles gambiae*, *Anopheles dirus*, and *Anopheles albimanus*. Antibodies against Pfs47 domain 2 (D2) have significant transmission-blocking activity that prevents *Plasmodium* ookinete development and is independent of human complement. Strong transmission-blocking activity has been mapped to a region of 52 amino acids in Pfs47 D2. Efforts to optimize the immunogenicity of the Pfs47 D2 antigen with a viral-like particle have been successful, and the efficacy of a P47-based TBV was confirmed in vivo with Pbs47, the orthologue of Pfs47 in the mouse malaria parasite *Plasmodium berghei*. The current evidence warrants further development and clinical testing of a Pfs47-based TBV.

## INTRODUCTION

The past two decades saw major advancements in alleviating malaria burden, primarily by reducing disease transmission through massive distribution of insecticide-treated nets and deployment of residual insecticide spraying. However, progress has recently stalled because of the emergence of insecticide resistance by malaria vectors and parasite resistance to antimalarial drugs,^1^ making more evident the need for new strategies to curb this devastating parasitic disease. Transmission-blocking vaccines (TBVs) are a potential new tool to assist in malaria elimination^2^ by eliciting a human immune response that prevents parasite development in the mosquito vector.^3^

Malaria TBVs take advantage of the *Plasmodium* parasite’s vulnerability as it emerges from red blood cells in the mosquito gut to engage in a complex developmental cycle that results in a natural life cycle bottleneck. Malaria transmission begins when a mosquito vector takes a blood meal containing *Plasmodium* gametocytes. Within minutes, these sexual-stage parasites mature into gametes, leave the red blood cell, and fuse, forming a fertilized zygote that matures into a motile ookinete. Usually, five or fewer ookinetes traverse the mosquito gut successfully and develop into the oocyst stage. The parasite multiplies continuously during the oocyst stage, and mature oocysts rupture and release thousands of sporozoites that invade the mosquito salivary gland. Sporozoites are infectious to humans, and they are injected into the skin of a new host when an infected mosquito takes an additional blood meal.^4^ It is therefore attractive to stop *Plasmodium* infection in the mosquito before the parasite multiplies during the oocyst stage.

## Pfs47 STRUCTURAL ORGANIZATION

*Plasmodium falciparum* sexual-stage surface protein 47 (Pfs47) is a predicted glycosylphosphatidylinositol (GPI) anchored protein of the *Plasmodium falciparum* six-cysteine (6-Cys) family, which includes two leading TBV targets: Pfs48/45 and Pfs230. This family comprises proteins with 1 to 14 cysteine-rich domains. The current 14 members of the 6-Cys family include secreted or membrane-anchored proteins that are involved in cell-to-cell interactions during fertilization, liver invasion, or mosquito immune evasion.^5^ Pfs48/45 and Pfs230 are expressed in gametocytes and on the surface of gametes, and are critical for male fertility.^6^^–^^8^ They both were initially recognized as TBV targets using transmission-blocking monoclonal antibodies (mAbs) raised against gametes.^9^ Pfs230 has 14 protein domains, each with a conserved pattern of cysteine residues.^10^ The other 6-Cys family members were later identified searching the canonical cysteine pattern on novel protein sequences of the *Plasmodium* genome. A correct prediction of the cysteine pairs that form the disulfide bonds and of the folding in the 6-Cys domain^11^ was followed by accurate computational modeling^12^ and experimental confirmation.^13^

**Figure 1.**
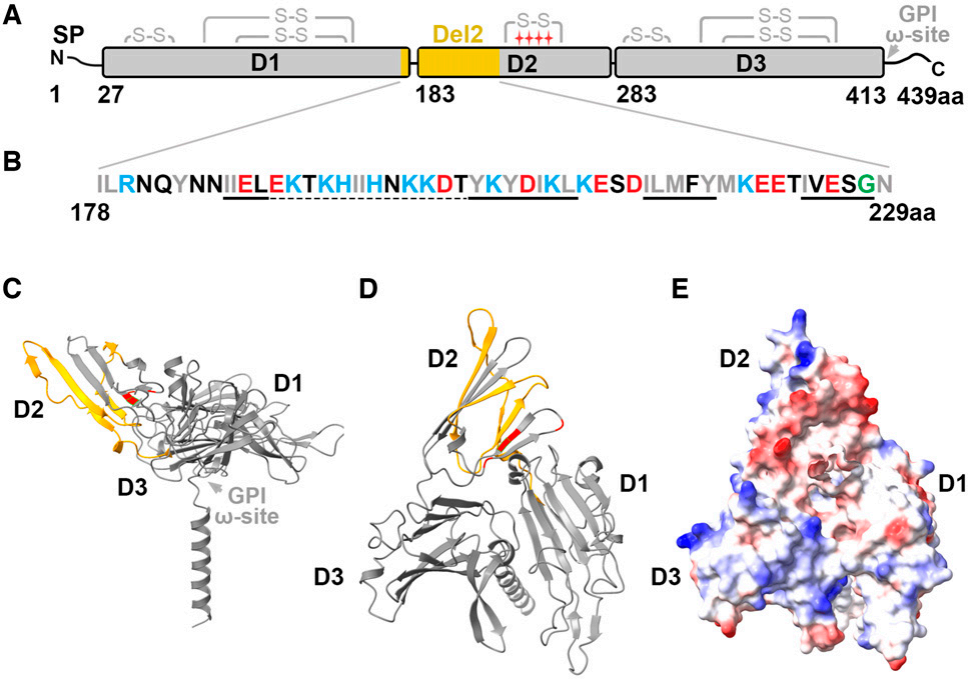
Pfs47 protein structure and the identified transmission-blocking vaccine (TBV) antigen Pfs47-Del2. (**A**) Schematic of Pfs47 drawn to scale shows Pfs47 three domains (D1, D2, D3), predicted disulfide bonds (S-S), and TBV antigen Pfs47-Del2 location (178–229 amino acid residue [aa], in yellow). Pfs47 D1 and D3 have the canonical cysteine arrangement of a six-cysteine domain, whereas D2 has only two cysteines. The approximate beginning of each protein domain is indicated by its aa number. The location of the aa polymorphisms that are known to determine *Plasmodium falciparum* compatibility to vectors is indicated with red stars. (**B**) Amino acid composition of TBV antigen Pfs47-Del2. The properties of the aa side chains are indicated by color: polar (black), nonpolar (gray), negatively charged (red) or positively charged (blue), and glycine (green). Predicted β-strands (–––) and disordered segments (- - - -) are indicated. The AlphaFold2-predicted Pfs47 three-dimensional structure is presented in a side view (**C**) and top views (**D, E**) without the signal peptide. The surface representation (**E**) shows negative (red) and positive (blue) charges. Pfs47-Del2 appears in yellow and the aa polymorphisms that determine *P. falciparum* compatibility to vectors are shown in red (**C, D**). GPI ω-site = predicted glycosylphosphatidylinositol anchoring site; SP = signal peptide.

Based on their similar gene structure and contiguous genomic location, Pfs47 and Pfs48/45 appear to have arisen from a gene duplication event, although they have low amino acid (aa) sequence identity (27%). Both Pfs47 and Pfs48/45 contain three protein domains: D1, D2, and D3. In Pfs47, D1 and D3 have the canonical 6-Cys pattern, whereas D2 is a shorter and degenerate s48/45 domain with only two cysteines (Figure 1A).^13^

The structure of Pfs47 was recently predicted using AlphaFold2,^14^ protein structure modeling software, and became publicly available (https://alphafold.ebi.ac.uk/entry/Q8IDN0). The software predicted most of the structure with confidence or high confidence (predicted local distance test, > 70 or > 90; predicted alignment error, 0.727). It predicts the presence of three domains with the canonical s48/45 domain conformation consisting of a β-sandwich formed by antiparallel and parallel β-sheets (Figure 1C and D).^13^ The approximate boundaries of the Pfs47 domains in the predicted structure are D1, aa 27 to 182; D2, aa 183 to 282; and D3, aa 283 to 415. The three Pfs47 domains are adjacent to each other, forming a triangular or “three-petal flower” arrangement (Figure 1D). The predicted GPI ω-site is toward the C-terminal of Pfs47 D3 (Figure 1C) presumably facing the cell membrane. Furthermore, D1 and D3 have very few negative charges (Figure 1E) on the surface facing away from the cell membrane, whereas this surface has many negative charges in D2 (Figure 1E, shown in red). The C-terminal of Pfs47 D2, containing the polymorphisms responsible for mosquito compatibility, is at the lower edge of the predicted β-sandwich (Figure 1C, in red). Multiple efforts to elucidate the structure of Pfs47 experimentally have not succeeded so far (M. Boulanger, personal communication).

## ROLE OF Pfs47 AND ITS ORTHOLOGUES IN *PLASMODIUM* FERTILIZATION AND MOSQUITO IMMUNE EVASION

Several functions have been attributed to Pfs47 and its orthologues. Pfs47 is expressed within *Plasmodium* female gametocytes, and on the surface of female gametes, and ookinetes.^15^^–^^17^ In vivo studies with *Plasmodium berghei*, a mouse malaria model, in which the *Pbs47* gene (the ortholog of *Pfs47*) was disrupted, revealed that Pbs47 is required for optimal female gamete fertility, as *Pb47* null parasites had lower gamete fusion and reduced infectivity in *Anopheles stephensi* Nijmegen (SDA 500) mosquitoes.^8^ However, laboratory infections with in vitro cultured gametocytes from a *P. falciparum*-*Pfs47* null line revealed that Pfs47 is not essential for female gamete fertility nor for infectivity in *An. stephensi* Nijmegen (SDA 500).^17^ Of note, this widely used laboratory mosquito strain is of Indian origin and was genetically selected to be highly susceptible to *P. falciparum* infection.^18^

Analysis of a genetic cross between two *P. falciparum* lines (GB4 and 7G8) that differ in their survival in *Anopheles gambiae* L3-5, led to the discovery that Pfs47 allows the malaria parasite to evade the mosquito complement-like system, a major final effector of mosquito anti-plasmodial immunity that targets the ookinete stage.^19^ Genetic mapping, linkage group selection,^20^ and functional genetics studies revealed that four aa polymorphisms between the two cysteines in Pfs47-D2 are key determinants of mosquito infectivity.^16^ Later studies showed that Pfs47 is required for efficient malaria transmission by several dominant anopheline vectors, including *An. gambiae* (Africa), *Anopheles dirus* (Southeast Asia) and *Anopheles albimanus* (New World).^16^^,^^21^ Pfs47 disrupts activation of a c-Jun N-terminal kinase/caspase-mediated apoptosis that involves a strong nitration response in the invaded midgut cell, preventing nitration of the subjacent basal lamina.^22^^,^^23^ Circulating mosquito hemocytes are attracted to the midgut by prostaglandin E2 release by the midgut in response to ookinete invasion.^24^ If hemocytes patrolling the midgut surface encounter a nitrated basal lamina, they undergo apoptosis and release microvesicles.^25^ Local release of hemocyte-derived microvesicles is required for effective activation of the mosquito complement-like system and for parasite elimination.^25^ Pfs47 allows *Plasmodium* immune evasion by disrupting midgut epithelial nitration, thus preventing local hemocyte-derived microvesicle release and rendering the mosquito complement system ineffective against the parasite. Pbs47 was as well found to be essential for ookinete evasion of the *An. gambiae* complement-like response.^26^

Several studies have shown that Pfs47 is highly polymorphic in Pfs47 D2 and exhibits a striking geographic population structure.^21^^,^^27^^,^^28^ Furthermore, direct experimental evidence showed that polymorphisms in Pfs47 D2 determine the compatibility of *P. falciparum* to *An. gambiae*, *An. dirus*, and *An. albimanus*, suggesting that selection of Pfs47 haplotypes was involved in the adaptation of *P. falciparum* to phylogenetically distant mosquito vectors.^21^ Pfs47 interacts with a receptor protein in the mosquito midgut, and the Pfs47 haplotype of a given parasite must be compatible with the receptor of the local vector, as a lock and key, for effective immune evasion that facilitates malaria transmission.^29^

## Pfs47 AS A TBV TARGET

Initial attempts to assess the potential of Pfs47 and its orthologue in *Plasmodium vivax* (Pvs47) as TBV targets had variable success. Three rat mAbs against Pfs47 had no significant effect on *P. falciparum* infection in standard membrane feeding assays (SMFAs) using in vitro cultured *P. falciparum* gametocytes to infect *An. stephensi* Nijmegen females.^17^ In contrast, DNA vaccine immunization of mice against full-length Pvs47 generated antisera with significant transmission blocking in *An. dirus*. This activity required human complement, in a direct membrane feeding assay with *P. vivax* gametocytes obtained directly from patients with malaria.^30^

The discovery of the key role of Pfs47 in the evasion of mosquito immunity, and the genetic evidence of the relevance of Pfs47 D2,^16^^,^^21^ led to a more systematic evaluation of Pfs47 as a TBV target. Soluble, full-length recombinant Pfs47, was expressed successfully as a fusion protein with thioredoxin (T-Pfs47) in *Escherichia coli*, and was immunogenic in mice. Pfs47 polyclonal antibodies recognized gametocytes and female gametes, but together with 14 Pfs47 mAbs had modest (< 65%) or no transmission-reducing activity (TRA) in SMFAs with *An. gambiae* G3 females—an unselected laboratory strain susceptible to *P. falciparum* infection. Interestingly, binding specificity analysis showed that antibodies after immunization with T-Pfs47 mostly recognized Pfs47 D1 or D3, but not D2.^15^ A similar study, in which mice were immunized with recombinant full-length Pfs47 ectodomains expressed in HEK293E cells, led to polyclonal antibodies with no gamete recognition by indirect fluorescent antibody assay and no transmission-blocking activity.^31^

In contrast, immunization with a Pfs47 D2 recombinant peptide (aa 155–264), in which the two cysteines were mutated to alanine to facilitate expression, generated polyclonal and mAbs that recognized gametocytes and female gametes, and had significant transmission blocking (78%–99% TRA). mAbs and deletion analysis identified a 52-aa region mostly in D2 (Pfs47-Del2, aa 178–229), immediately preceding the predicted Pfs47 D2 disulfide bond, where antibody binding disrupts malaria transmission (Figure 1A, yellow). This target region is highly conserved (96%–98% aa identity) in parasites from different geographic regions, with only seven unique haplotypes that mostly differ by a single aa.^15^ Further studies are needed to test with heterologous parasites the cross-reactivity and transmission-blocking activity of sera developed against the most frequent Pfs47 haplotype in Africa (GB4).

The higher immunogenicity of Pfs47 D1 and D3, which generate non-transmission-blocking antibodies, suggests that they may act as antigenic decoys to prevent antibody generation against functional regions in Pfs47 D2. The predicted three-dimensional structure of Pfs47 (Figure 1B–D) indicates that Pfs47 D2 is accessible to antibodies. The lower immunogenicity of Pfs47 D2 coincides with higher negative surface charge, and a reduced number of cysteines (one pair), compared with Pfs47 D1 and D3 (three pairs), which could make Pfs47 D2 conformation more flexible and less immunogenic, and may also explain the difficulty in obtaining a Pfs47 crystal structure.

Transmission-blocking mAb IB2 reduced the number of ookinetes in the mosquito midgut lumen independent of human complement, suggesting that IB2 blocks gamete fertilization or ookinete development by steric hindrance. Although Pfs47 appears not to be essential for fertilization, since Pfs47 null gametocytes infect *An. stephensi* Nijmegen females.^17^ However, mosquitoes are fed large numbers of gametocytes in SMFAs, so that even if the process was less efficient for the Pfs47 null parasite, enough fertilization events may still occur. IB2 has strong TRA in both *An. gambiae* G3 and *An. stephensi* Nijmegen, and disruption of the mosquito complement system in *An. gambiae* G3 did not affect the TRA, suggesting that it is also independent of mosquito complement.^15^ In contrast, mAb JH11, which binds to Pfs47 D1 close to the boundary with Pfs47 D2, increased consistently and significantly the number of midgut oocysts in SMFA.^15^ The effect of mAb JH11 may be a result of stabilization of a conformation that facilitates the interaction between gametes during fertilization or between Pfs47 with its mosquito receptor.

Interestingly, potent transmission-blocking antibodies against Pfs48/45 and Pfs230 are directed mainly to a specific domain—similar to observations with Pfs47. Although some Pfs48/45 mAbs that bind to epitopes in D2 and D3 reduce transmission,^32^^,^^33^ antibodies with the strongest transmission blocking activity target Pfs48/45 D3,^34^ a domain with six cysteines. To date, only antibodies that target Pfs230 D1 (aa 589–730, with four cysteines) are effective.^35^

However, there are important differences in the architecture of their target domains and the mechanism of transmission blocking of antibodies against these three 6-Cys proteins. The TRA of both Pfs48/45 and Pfs47 antibodies does not require human complement. In contrast, human complement greatly enhances the TRA of most antibodies that target Pfs230.^36^ Pfs47 mAbs identified so far with strong transmission-blocking activity recognize reduced Pfs47 protein, indicating that they bind to linear epitopes^15^; however, most transmission-blocking antibodies that target Pfs48/45 and Pfs230 only recognize their target proteins when their disulfide bonds are intact (non-reduced state), suggesting they bind to conformational epitopes.^9^^,^^32^^,^^36^^,^^37^ Pfs230 mAb 4F12 is an interesting exception because it has been shown to recognize both native and denatured Pfs230, and it has effective transmission blocking in the absence of human complement.^38^ The antibodies that do not require human complement for transmission blocking may disrupt domain structure and/or block access to interacting proteins.

The Pfs47-Del2 antigen, which generates antibodies that block transmission, forms a disordered segment toward its N-terminal region (E190-T202) followed by three β-strands of the β-sandwich in Pfs47 D2, according to the AlphaFold2-predicted Pfs47 structure (Figure 1B–D). Positively charged aa’s predominate in the disordered region, which is followed by a region where non-polar and negatively charged aa’s predominate (Figure 1B).

Individuals in malaria-endemic areas generate antibodies against Pfs47^39^; however, no association has been established between transmission-blocking activity and natural anti-Pfs47 antibody levels.^40^ This is not surprising, as we have found that antibodies to the most immunogenic Pfs47 domains (D1 and D3) do not block disease transmission.

The efficacy of a P47-based TBV was tested in vivo with *P. berghei*. Full-length recombinant Pbs47 was expressed in *E. coli* as a fusion protein with thioredoxin. Immunization with full-length Pbs47 generated antibodies that mostly recognized Pbs47 D1 and Pbs47 D3, but not Pbs47 D2, and had no significant transmission blocking in direct mosquito feeding on mice—similar to vaccinating with full-length Pfs47. Furthermore, a region of Pbs47 D2 similar to Pfs47-Del2 (Pbs47-Del1, with the two cysteines substituted for alanine) generated polyclonal antibodies with significant TRA in both active and passive immunization assays, with IgG concentrations as low as 50 μg/mL in mouse serum. Affinity-purified Pbs47-Del1rabbit antibodies had strong TRA (> 80%) after passive immunization in mice with 1 μg/mL IgG,^41^ suggesting that increasing the immunogenicity of the Pfs47 antigen may lead to high transmission blocking in vivo.

## OPTIMIZATION OF A Pfs47-BASED TBV

In early experiments, immunization with Pfs47-Del2 peptide together with a cytosine phosphoguanine (CpG)-based adjuvant (Magic Mouse^©^, Creative Diagnostics, Shirley, NY) generated antibodies with significant and reproducible transmission-blocking activity (> 89% TRA) at a concentration of 200 μg/μL IgG.^15^ A 58-aa Pfs47 target antigen (aa 178–235) that includes Pfs47-Del2 was then linked to a virus-like particle (VLP) using the AP205-SpyCatcher:SpyTag platform system to enhance immune recognition and increase antibody titers.^42^ Of the different antigen combinations tested, sera obtained after an initial immunization with VLP-Pfs47 followed by a boost with Pfs47 peptide gave the highest transmission blocking, requiring antibody concentrations as low as 5 μg/μL IgG in SMFA.^43^ However, binding specificity tests indicated that the majority of the antibodies produced were directed to the virus portion of the VLP and not the Pfs47 antigen^43^; thus, other platforms are being tested to optimize Pfs47 antigen presentation further (unpublished results).

The potential of delivering the relatively small (52–58 aa) Pfs47 antigen using microneedle technology was explored, because this vaccine delivery platform offers substantial advantages.^44^ Antigen-loaded dissolving microneedles (DMNs) are micrometer-size structures that can be applied to the skin in a Band-Aid-like fashion. This avoids the need for a needle injection and could, in principle, be kept at room temperature for an extended time, simplifying vaccine storage and delivery under field conditions. Gelatin-based microneedles containing Pfs47 antigen, and CpG (toll-like receptor 9 agonist) as an adjuvant, were manufactured successfully and tested for stability and solubility. The DMNs punctured the mouse ear skin and dissolved efficiently. The Pfs47 antigen in DMNs showed no signs of degradation after 1 week of storage at room temperature. The CpG adjuvant in the Pfs47 DMNs was also active, and stimulated toll-like receptor 9 signaling in reporter cells and splenic dendritic cells.^45^ The in vivo immunogenicity of Pfs47 DMNs remains to be established.

## CONCLUSION

Further development and clinical testing of a Pfs47-based malaria TBV is called for, based on the strong experimental evidence that antibody binding to a specific region of Pfs47 blocks malaria transmission and that this target region is immunogenic. At present, it is not known whether naturally acquired anti-Pfs47 antibodies are protective nor whether antibodies to immuno-dominant domains, which do not block transmission, limit the access to Pfs47 D2 epitopes where antibody binding could disrupt transmission. It is also not clear whether a Pfs47 D2-based vaccine would be boosted by natural malaria infections. Because the transmission blocking mechanism of the Pfs47 target antigen appears to be independent from that of other TBV targets, testing whether this antigen could synergize when combined with other vaccine antigens is warranted.

## References

[1] World Health Organization , 2021. World Malaria Report 2021. Geneva, Switzerland: WHO.

[2] Alonso PL 2011. A research agenda to underpin malaria eradication. PLoS Med 8: e1000406. 21311579 10.1371/journal.pmed.1000406 PMC3026687

[3] Duffy PE , 2021. Transmission-blocking vaccines: harnessing herd immunity for malaria elimination. Expert Rev Vaccines 20: 185–198. 33478283 10.1080/14760584.2021.1878028 PMC11127254

[4] Smith RC Vega-Rodriguez J Jacobs-Lorena M , 2014. The *Plasmodium* bottleneck: malaria parasite losses in the mosquito vector. Mem Inst Oswaldo Cruz 109: 644–661. 25185005 10.1590/0074-0276130597 PMC4156458

[5] Arredondo SA Kappe SH , 2016. The s48/45 six-cysteine proteins: mediators of interaction throughout the *Plasmodium* life cycle. Int J Parasitol 47: 409–423. 27899328 10.1016/j.ijpara.2016.10.002 PMC5446806

[6] Eksi S Czesny B Van Gemert G-J Sauerwein RW Eling W Williamson KC , 2006. Malaria transmission-blocking antigen, Pfs230, mediates human red blood cell binding to exflagellating male parasites and oocyst production. Mol Microbiol 61: 991–998. 16879650 10.1111/j.1365-2958.2006.05284.x

[7] van Dijk MR 2001. A central role for P48/45 in malaria parasite male gamete fertility. Cell 104: 153–164. 11163248 10.1016/s0092-8674(01)00199-4

[8] van Dijk MR 2010. Three members of the 6-cys protein family of *Plasmodium* play a role in gamete fertility. PLoS Pathog 6: e1000853. 20386715 10.1371/journal.ppat.1000853 PMC2851734

[9] Quakyi IA Carter R Rener J Kumar N Good MF Miller LH , 1987. The 230-kDa gamete surface protein of *Plasmodium falciparum* is also a target for transmission-blocking antibodies. J Immunol 139: 4213–4217. 2447164

[10] Williamson KC Criscio MD Kaslow DC , 1993. Cloning and expression of the gene for *Plasmodium falciparum* transmission-blocking target antigen, Pfs230. Mol Biochem Parasitol 58: 355–358. 8479460 10.1016/0166-6851(93)90058-6

[11] Carter R Coulson A Bhatti S Taylor BJ Elliott JF , 1995. Predicted disulfide-bonded structures for three uniquely related proteins of *Plasmodium falciparum*, Pfs230, Pfs48/45 and Pf12. Mol Biochem Parasitol 71: 203–210. 7477102 10.1016/0166-6851(94)00054-q

[12] Gerloff DL Creasey A Maslau S Carter R , 2005. Structural models for the protein family characterized by gamete surface protein Pfs230 of *Plasmodium falciparum.* Proc Natl Acad Sci USA 102: 13598–13603. 16155126 10.1073/pnas.0502378102 PMC1224620

[13] Arredondo SA Cai M Takayama Y MacDonald NJ Anderson DE Aravind L Clore GM Miller LH , 2012. Structure of the *Plasmodium* 6-cysteine s48/45 domain. Proc Natl Acad Sci USA 109: 6692–6697. 22493233 10.1073/pnas.1204363109 PMC3340019

[14] Jumper J 2021. Highly accurate protein structure prediction with AlphaFold. Nature 596: 583–589. 34265844 10.1038/s41586-021-03819-2 PMC8371605

[15] Canepa GE 2018. Antibody targeting of a specific region of Pfs47 blocks *Plasmodium falciparum* malaria transmission. NPJ Vaccines 3: 26. 30002917 10.1038/s41541-018-0065-5 PMC6039440

[16] Molina-Cruz A 2013. The human malaria parasite Pfs47 gene mediates evasion of the mosquito immune system. Science 340: 984–987. 23661646 10.1126/science.1235264 PMC3807741

[17] van Schaijk BC 2006. Pfs47, paralog of the male fertility factor Pfs48/45, is a female specific surface protein in *Plasmodium falciparum.* Mol Biochem Parasitol 149: 216–222. 16824624 10.1016/j.molbiopara.2006.05.015

[18] Feldmann AM Ponnudurai T , 1989. Selection of *Anopheles stephensi* for refractoriness and susceptibility to *Plasmodium falciparum.* Med Vet Entomol 3: 41–52. 2519646 10.1111/j.1365-2915.1989.tb00473.x

[19] Blandin S Shiao SH Moita LF Janse CJ Waters AP Kafatos FC Levashina EA , 2004. Complement-like protein TEP1 is a determinant of vectorial capacity in the malaria vector *Anopheles gambiae* . Cell 116: 661–670. 15006349 10.1016/s0092-8674(04)00173-4

[20] Pattaradilokrat S Cheesman SJ Carter R , 2007. Linkage group selection: towards identifying genes controlling strain specific protective immunity in malaria. PLoS One 2: e857. 17848988 10.1371/journal.pone.0000857 PMC1959240

[21] Molina-Cruz A Canepa GE Kamath N Pavlovic NV Mu J Ramphul UN Ramirez JL Barillas-Mury C , 2015. *Plasmodium* evasion of mosquito immunity and global malaria transmission: the lock-and-key theory. Proc Natl Acad Sci USA 112: 15178–15183. 26598665 10.1073/pnas.1520426112 PMC4679011

[22] Garver LS de Almeida Oliveira G Barillas-Mury C , 2013. The JNK pathway is a key mediator of *Anopheles gambiae* antiplasmodial immunity. PLoS Pathog 9: e1003622. 24039583 10.1371/journal.ppat.1003622 PMC3764222

[23] Ramphul UN Garver LS Molina-Cruz A Canepa GE Barillas-Mury C , 2015. *Plasmodium falciparum* evades mosquito immunity by disrupting JNK-mediated apoptosis of invaded midgut cells. Proc Natl Acad Sci USA 112: 1273–1280. 25552553 10.1073/pnas.1423586112 PMC4321252

[24] Barletta ABF Trisnadi N Ramirez JL Barillas-Mury C , 2019. Mosquito midgut prostaglandin release establishes systemic immune priming. iScience 19: 54–62. 31351392 10.1016/j.isci.2019.07.012 PMC6661395

[25] Castillo JC Ferreira ABB Trisnadi N Barillas-Mury C , 2017. Activation of mosquito complement antiplasmodial response requires cellular immunity. Sci Immunol 2: eaal1505. 28736767 10.1126/sciimmunol.aal1505 PMC5520810

[26] Ukegbu CV Giorgalli M Yassine H Ramirez JL Taxiarchi C Barillas-Mury C Christophides GK Vlachou D , 2017. *Plasmodium berghei* P47 is essential for ookinete protection from the *Anopheles gambiae* complement-like response. Sci Rep 7: 6026. 28729672 10.1038/s41598-017-05917-6 PMC5519742

[27] Anthony TG Polley SD Vogler AP Conway DJ , 2007. Evidence of non-neutral polymorphism in *Plasmodium falciparum* gamete surface protein genes Pfs47 and Pfs48/45. Mol Biochem Parasitol 156: 117–123. 17826852 10.1016/j.molbiopara.2007.07.008

[28] Manske M 2012. Analysis of *Plasmodium falciparum* diversity in natural infections by deep sequencing. Nature 487: 375–379. 22722859 10.1038/nature11174 PMC3738909

[29] Molina-Cruz A 2020. Plasmodium falciparum evades immunity of anopheline mosquitoes by interacting with a Pfs47 midgut receptor. Proc Natl Acad Sci USA 117: 2597–2604. 31969456 10.1073/pnas.1917042117 PMC7007573

[30] Tachibana M 2015. *Plasmodium vivax* gametocyte proteins, Pvs48/45 and Pvs47, induce transmission-reducing antibodies by DNA immunization. Vaccine 33: 1901–1908. 25765968 10.1016/j.vaccine.2015.03.008

[31] Nikolaeva D 2020. Functional characterization and comparison of *Plasmodium falciparum* proteins as targets of transmission-blocking antibodies. Mol Cell Proteomics 19: 155–166. 29089373 10.1074/mcp.RA117.000036 PMC6944241

[32] Carter R Graves PM Keister DB Quakyi IA , 1990. Properties of epitopes of Pfs 48/45, a target of transmission blocking monoclonal antibodies, on gametes of different isolates of *Plasmodium falciparum.* Parasite Immunol 12: 587–603. 1707506 10.1111/j.1365-3024.1990.tb00990.x

[33] Targett G , 1990. Immunity to sexual stages of human malaria parasites: immune modulation during natural infections, antigenic determinants, and the induction of transmission-blocking immunity. Scand J Infect Dis Suppl 76: 79–88. 1714627

[34] Roeffen W Teelen K van As J vd Vegte-Bolmer M Eling W Sauerwein R , 2001. *Plasmodium falciparum*: production and characterization of rat monoclonal antibodies specific for the sexual-stage Pfs48/45 antigen. Exp Parasitol 97: 45–49. 11207113 10.1006/expr.2000.4586

[35] Tachibana M 2019. Identification of domains within Pfs230 that elicit transmission blocking antibody responses. Vaccine 37: 1799–1806. 30824357 10.1016/j.vaccine.2019.02.021 PMC6708081

[36] Read D Lensen AH Begarnie S Haley S Raza A Carter R , 1994. Transmission-blocking antibodies against multiple, non-variant target epitopes of the *Plasmodium falciparum* gamete surface antigen Pfs230 are all complement-fixing. Parasite Immunol 16: 511–519. 7532850 10.1111/j.1365-3024.1994.tb00305.x

[37] Outchkourov N 2007. Epitope analysis of the malaria surface antigen pfs48/45 identifies a subdomain that elicits transmission blocking antibodies. J Biol Chem 282: 17148–17156. 17426022 10.1074/jbc.M700948200

[38] MacDonald NJ 2016. Structural and immunological characterization of recombinant 6-cysteine domains of the *Plasmodium falciparum* sexual stage protein Pfs230. J Biol Chem 291: 19913–19922. 27432885 10.1074/jbc.M116.732305 PMC5025679

[39] Paul NH Vengesai A Mduluza T Chipeta J Midzi N Bansal GP Kumar N , 2016. Prevalence of *Plasmodium falciparum* transmission reducing immunity among primary school children in a malaria moderate transmission region in Zimbabwe. Acta Trop 163: 103–108. 27491342 10.1016/j.actatropica.2016.07.023 PMC5007214

[40] de Jong RM Tebeje SK Meerstein-Kessel L Tadesse FG Jore MM Stone W Bousema T , 2020. Immunity against sexual stage *Plasmodium falciparum* and *Plasmodium vivax* parasites. Immunol Rev 293: 190–215. 31840844 10.1111/imr.12828 PMC6973022

[41] Yenkoidiok-Douti L Canepa GE Barletta ABF Barillas-Mury C , 2020. In vivo characterization of *Plasmodium berghei* P47 (Pbs47) as a malaria transmission-blocking vaccine target. Front Microbiol 11: 1496. 32719666 10.3389/fmicb.2020.01496 PMC7348136

[42] Brune KD Leneghan DB Brian IJ Ishizuka AS Bachmann MF Draper SJ Biswas S Howarth M , 2016. Plug-and-display: decoration of virus-like particles via isopeptide bonds for modular immunization. Sci Rep 6: 19234. 26781591 10.1038/srep19234 PMC4725971

[43] Yenkoidiok-Douti L Williams AE Canepa GE Molina-Cruz A Barillas-Mury C , 2019. Engineering a virus-like particle as an antigenic platform for a Pfs47-targeted malaria transmission-blocking vaccine. Sci Rep 9: 16833. 31727945 10.1038/s41598-019-53208-z PMC6856133

[44] Kim Y-C Park J-H Prausnitz MR , 2012. Microneedles for drug and vaccine delivery. Adv Drug Deliv Rev 64: 1547–1568. 22575858 10.1016/j.addr.2012.04.005 PMC3419303

[45] Yenkoidiok-Douti L Barillas-Mury C Jewell CM , 2021. Design of dissolvable microneedles for delivery of a Pfs47-based malaria transmission-blocking vaccine. ACS Biomater Sci Eng 7: 1854–1862. 33616392 10.1021/acsbiomaterials.0c01363 PMC8113916

